# Supplementary material for: Regulatory architecture controlling terminal differentiation of an interoceptive paraneuron in Caenorhabditis elegans
Source: Development. 2026 Sep 3;153(17):dev205749. doi: 10.1242/dev.205749 (PMC13615684; doi:10.1242/dev.205749)
Supplement: Supplementary file 1 [file develop-153-205749-s1.pdf]

Table S1. Strains used

Available for download at
https://journals.biologists.com/dev/article-lookup/doi/10.1242/dev.205749#supplementary-data

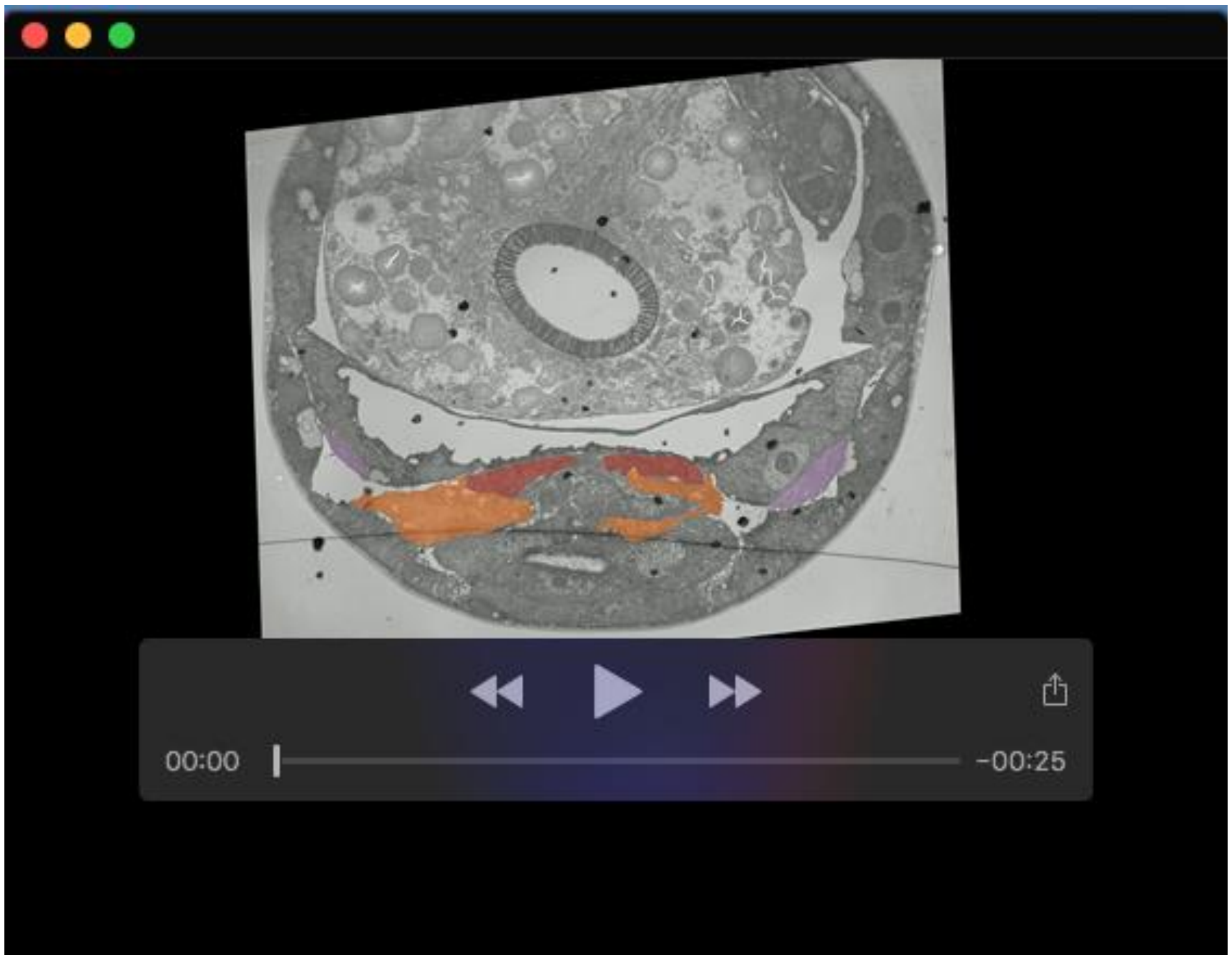

Movie 1. Segmented volumetric image stack of electron microscopy data from the vulval region of a late L4 *C. elegans* hermaphrodite. Green = HSNs, Purple = uv1s, Blue = VC4 and VC5, orange = vm1, red = vm2.

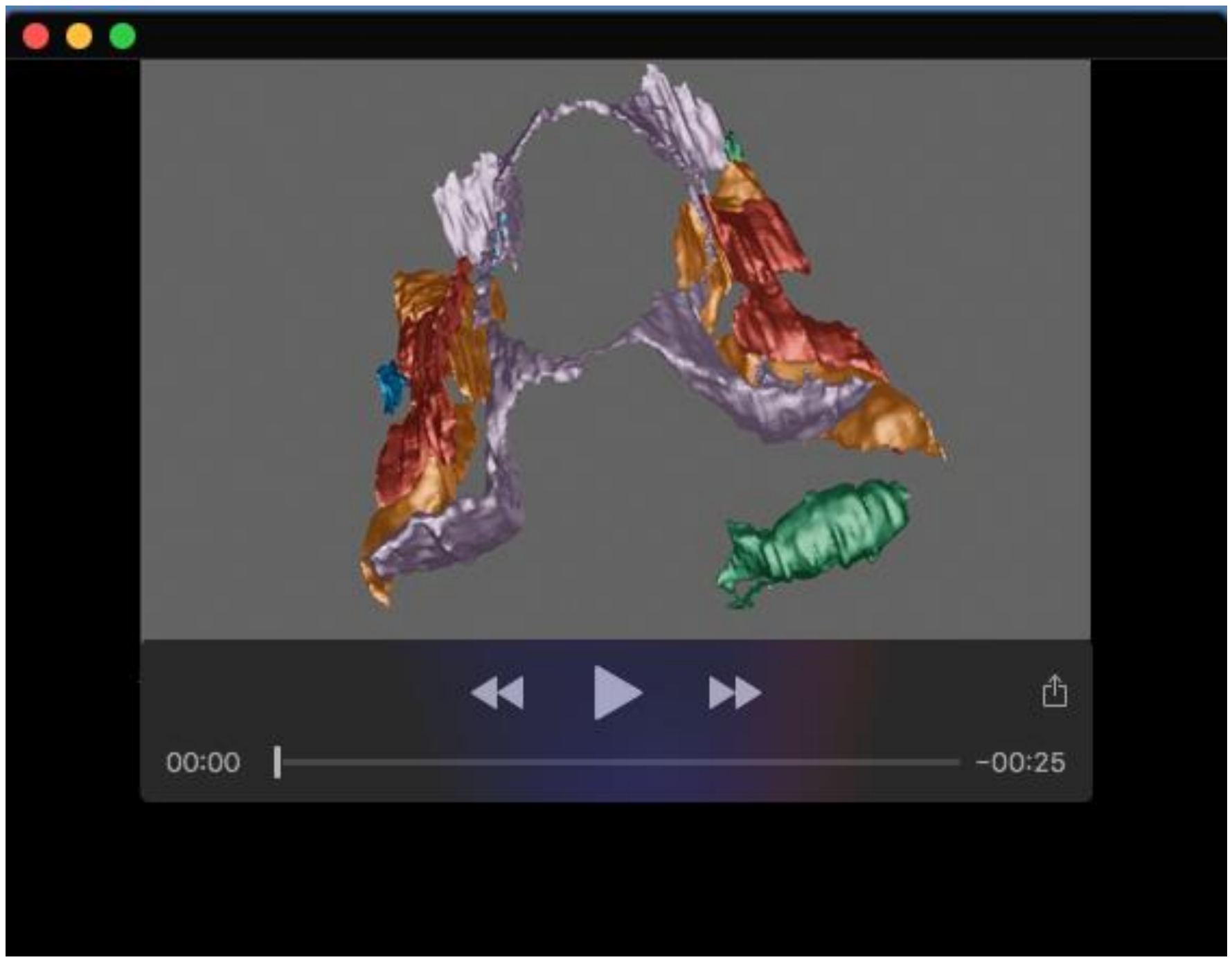

Movie 2. 3D reconstruction from electron microscopy data of vulvar anatomy of an L4 *C. elegans* hermaphrodite. Green = HSNL and HSNR, Purple = uv1, Blue = VC4 and VC5, orange = vm1, red = vm2.

Development • Supplementary information
