# Supplementary material for: Regulatory architecture controlling terminal differentiation of an interoceptive paraneuron in Caenorhabditis elegans
Source: Development. 2026 Sep 3;153(17):dev205749. doi: 10.1242/dev.205749 (PMC13615684; doi:10.1242/dev.205749)
Supplement: Supplementary file 2 [file develop-153-205749_review_history.pdf]

Regulatory architecture controlling terminal differentiation of an interoceptive paraneuron in *Caenorhabditis elegans*

Hongzhu Ji, Berta Vidal, Elisabeth Conklin, Tergel Enkhtuvshin, Nathan E. Schroeder and Oliver Hobert
DOI: 10.1242/dev.205749

Editor: Swathi Arur

Review timeline
Original submission: 20 April 2026
Editorial decision: 19 May 2026
First revision received: 2 July 2026
Accepted: 17 July 2026

Original submission

First decision letter

MS ID#: dev.205749

MS TITLE: Regulatory architecture controlling terminal differentiation of an interoceptive paraneuron in C. elegans

AUTHORS: Hongzhu Ji; Berta Vidal; Elisabeth Conklin; Tergel Enkhtuvshin; Nathan E. Schroeder; Oliver Hobert

Dear Dr Hobert,

I have now received all the referees' reports on the above manuscript, and have reached a decision. The referees' comments are appended below.

As you will see, the referees express considerable interest in your work, but have some significant criticisms and recommend a substantial revision of your manuscript before we can consider publication. If you are able to revise the manuscript along the lines suggested, which may involve further experiments, I will be happy receive a revised version of the manuscript. Your revised paper will be re-reviewed by one or more of the original referees, and acceptance of your manuscript will depend on your addressing satisfactorily the reviewers' major concerns. Please also note that Development will normally permit only one round of major revision.

Please attend to all of the reviewers' comments and ensure that you upload both a 'clean' version of your Word file, along with a highlighted version clearly showing where you have made changes in the revised manuscript. Please avoid using 'Tracked changes' in Word files as these are lost in PDF conversion. I should be grateful if you would also provide a point-by-point response detailing how you have dealt with the points raised by the reviewers in the 'Response to Reviewers' box. If you do not agree with any of their criticisms or suggestions please explain clearly why this is so.

Reviewer 1

SUMMARY OF THE ADVANCE MADE IN THIS PAPER AND ITS POTENTIAL SIGNIFICANCE TO THE FIELD

© 2026. Published by The Company of Biologists under the terms of the Creative Commons Attribution License (https://creativecommons.org/licenses/by/4.0/).

In this manuscript, the authors document the expression of genes that are expected to be neuronally specific to a set of cells not normally considered neurons in C. elegans. These uv1 cells are part of the egg-laying circuitry and are known to be activated in a mechanosensory fashion. Overall, I find this to be a well written and thoughtful analysis of these cells. I agree with the authors' statement that cells like uv1 and other "paraneurons" do raise intriguing questions about what the definition of a neuron is. In this case, I am curious what the authors think that is. I look at the fact that the cells sense mechanosensory (compression) signals, are secretory, with some (much?) of the exocytotic machinery found in neurons, and these cells facilitate a neuronally-induced behavior (egg-laying). While most neurons in most organisms are largely derived from ectodermal lineages, there are obvious examples of exceptions. In C. elegans there are other neurons derived from the MS lineage, which also generate the uv1 cell types. In addition, the reconstruction of the uv1 cells here demonstrate neuronal-like processes that contain vesicles.

Overall, I would encourage the authors to provide a bit more clarity on what separates neurons from paraneurons in the introduction to the paper. Certainly, there are more "secretory" neurons in higher mammals that operate in a fashion similar to uv1, rather than evoked release of chemical neurotransmitters.

I have few concerns about the manuscript. The experiments are well done, appropriate controls are in place, and the quantification, statistical analyses, and presentation are all rigorous. While the overall N number of animals observed in Figure 2 is lower than we might typically see in C. elegans papers, the effects are binary, and thus, highly unlikely to be spurious.

I support this paper for inclusion in Development, specifically I see the advance here as a very thorough genetic dissection of the differentiation of an understudied cell type in C. elegans. The relative question about paraneurons versus neurons should be strengthened to highlight the innovative nature of this, but I believe that can be done with additional information from the authors.

SUGGESTIONS TO AUTHORS

Questions for the authors to address-
1) Can they please be more precise about what features the uv1 cells share with paraneurons that are absent from neurons and/or vice versa? I am not opposed to calling these cells paraneurons, but, it would be helpful to have all of the key facts as we consider this terminology.
2) Can they please comment on the perdurance of the egl-3 expression in the HSNs in the CUT triple mutants (this may be relevant to my Q1).
3) Can the authors comment on whether any of the auxin depletion experiments resulted in the expression of markers that enabled them to visualize uv1, but led to morphological errors? Specifically, are these terminal selectors responsible for the neuron-like processes that these cells form? It may be possible to see that in an "incomplete" depletion of the protein.
Suggestions I would ask that the authors add the N values for Fig2B and in Figure 3 in the columns as was done for each of the other graphs. I appreciate that the points are added as a scatterplot on top of the bars, but the overlap puts the onus of counting them on the reader.
Please describe briefly in the text when during development the uv1 cells form, this will be helpful for interpreting the auxin experiments. For example, a graphic timeline of the experiments in Figure 3, that is, when, relative to the formation of the uv1 cells is auxin applied and when are the markers evaluated?
Note, I am not concerned about the "intermediate" effect of depletion, given the genetic loss of function data, but it is worth considering that some earlier events are lin-11 and/or egl-38 dependent and that those events can compensate for later depletion of those proteins.
I would encourage the authors to present a table of the markers used and the terminal selectors being manipulated (genetically and by auxin). It would be helpful to note the category of proteins that are present.

Reviewer 2

SUMMARY OF THE ADVANCE MADE IN THIS PAPER AND ITS POTENTIAL SIGNIFICANCE TO THE FIELD

© 2026. Published by The Company of Biologists under the terms of the Creative Commons Attribution License (https://creativecommons.org/licenses/by/4.0/).

The study provides evidence for a set of transcription factors to be involved in cell fate specification of uv1 cells in C. elegans using a battery of cell fate markers. The identified transcription factors are also involved in the specification of neuronal cell fate providing support for the hypothesis that paraneurons and neurons may be sharing evolutionary origins. Therefore, the advance to the field is the description of uv1 cells as paraneurons in the nematode C. elegans and the shared mechanisms of cell fate specification. Overall, this is an interesting concept and contributes to our understanding of the specification of paraneurons in general and specifically in C. elegans. Current shortcomings include the fact that the new findings have not been analyzed or discussed in the context of what is known about cell fate specification of paraneurons more generally.

SUGGESTIONS TO AUTHORS

In this paper Ji and colleagues describe the morphology of the uv1 cells in C elegans, which the authors describe as paraneurons, a term coined in the 70ies. These cells share sets of genes and neuroendocrine functions, but lack the classic morphology of neurons. The authors present an argument that the uv1 cells should be considered paraneurons because they are (1) derived from epithelial cells, (2) sense pressure in the uterus and (3) secrete neuropeptides. The authors then go on to show expression of several cell fate markers (common in neurons), and describe how these cell fate markers are under the control of a set of transcription factors previously known to specify neuronal cell fate. Overall, the study is solid and potentially interesting in that it sheds light on the specification of paraneurons. The manuscript is well written and, strengths include the sophisticated reporters although these reporters do not lead to much different findings than the previously reported reporters. The study also inducible loss of function alleles. These allele show convincingly (what previously loss of function alleles could not) that the tested transcription factor genes are required for the specification of uv1 cells and for maintenance of cell fate. As is, the manuscript reads a bit wormy and no attempt is made to place the findings into a larger context of what is known about the specification of paraneurons more generally. For example, basic HLH are one group of transcriptional regulators that appears to specify paraneurons across phyla [PMID: 41253114], yet no attempt is made to either test or discuss them in the context of the current findings. Placing the current findings into a larger context could significantly increase the appeal to a broader readership.

Points to be addressed:

1. Do mutations in bHLH genes such as lin-32/atoh or others show phenotypes in uv1, either in terms of cell fate or morphology?
2. The authors describe a number of similarities between the uv1 paraneurons and neurons in terms of gene expression programs. Where are the differences between paraneurons and neurons in terms of their specification?
3. The authors show that reporters for lin-11, egl-38 and egl-13 are expressed uv1 (as was shown before), leading them to state that "lin-11, egl-38 and egl-13 [] define a unique combinatorial signature of transcription factor[s]". It is not clear why this is a unique signature. Because this combination has never been seen in any other cell?
4. At least some of the transcription factor mutants tested show expression of cell fate markers in putative uv1 cells, raising the possibility that in at least a subset of animals uv1 morphology could be investigated using the reporters (such as sri-5 or ida-1).
5. The authors state that CEH-44 and CEH-48 are expressed in uv1. Are the reporters used in Fig.1E transcriptional reporters or protein fusions? Depending on the answer to this question (the strains are not described or referenced), the authors should refer to them as markers for expression of these genes.
6. What is the evidence that the circled cells in Fig.1D-F are uv1 cells? Is position sufficiently unambiguous?
7. p4: should read "First, we assessed expression of the panneuronal marker egl-3…"
1. There are numerous mistakes/omissions in the References
a. Collins et al. 2016 seems duplicated
b. Eroglu et al is incomplete
c. Hills-Muckey et al. is incomplete
d. Sepers et al. is incomplete
e. Young et al. is incomplete

© 2026. Published by The Company of Biologists under the terms of the Creative Commons Attribution License (https://creativecommons.org/licenses/by/4.0/).

Reviewer 3

SUMMARY OF THE ADVANCE MADE IN THIS PAPER AND ITS POTENTIAL SIGNIFICANCE TO THE FIELD

The manuscript from Ji and colleagues investigated how epithelial cells called uv1 cells develop neuronal features. These uv1 cells were described as neuroendocrine cells in previous studies but are considered paraneuronal cells in this manuscript due to their expression of neuronal genes involved with vesicular release and neuromodulatory functions. Understanding how non-neuronal precursors become bona fide neurons may provide insights into important cellular events that gave rise to neurons during evolution. Studies like these could also shed light onto how the fates of neurons and closely related cells are distinguished in multicellular organisms. To identify how candidate transcription factors influence the expression of neuronal genes in uv1 cells, the authors use fluorescent reporters in animals harboring mutations in three transcription factor genes. Based on these genetic experiments, the authors conclude that lin-11, egl-13, and egl-38 act in a combinatorial manner to coordinate the terminal differentiation of uv1 epithelial cells into paraneuronal cells that express genes associated with neuronal function. Despite excitement for this avenue of research, there are concerns regarding experimental analysis and conclusions drawn from the data provided in this manuscript. The authors identify neuronal genes regulated by these three transcription factors; however, the study provides no mechanistic insight into how they do so. The methods for measuring gene expression lack complete description of the analysis, including statistical analysis and how the authors reliably identified uv1 cells during the studies. The authors focus their conclusions on terminal differentiation. Yet, the importance placed on these events is overstated and over-simplified based on data collected from mutant animals, which display cell fate changes prior to terminal differentiation steps.

SUGGESTIONS TO AUTHORS

Major Concerns:

1. The authors conclude that neuronal genes are expressed in uv1 cells based on fluorescence-based reporters. The data show expression of nuclear localized reporters for genes of interest in a variable number of cells with variable placements within the animals. As presented, it's not clear how the authors identified the uv1 cells in these experiments. The authors show expression patterns of uv1 markers in Figure 1C; however, based on the strain list, these markers were not used to identify uv1 cells for the analyses in this study. How did the authors accurately identify uv1 cells in various mutant and degron expressing backgrounds?
2. The authors suggest non-stereotypical vesicles are present in uv1 cells. Based on the EM images, it is difficult to identify the vesicles even with the arrowheads marking two vesicles per cell. More information about the presence of vesicles would help support this conclusion. Do these vesicles appear in multiple sections? In other words, could these images represent cross sections of a larger structure that looks like a vesicle? Do vesicle markers localize to uv1 cells in these same regions? What is the frequency with which vesicles are observed in these cells?
3. The analysis by fluorescence microscopy is incompletely described. What stage(s) of animals were imaged? What are the details of the objective (magnification, immersion, numerical aperture, etc.)?
4. The authors created a novel null allele of lin-11, since the loss of function effects of previous alleles were deemed questionable. Since previously used loss of function alleles have shown varying effects on the phenotypes, a true null might be expected to present severe defects in vulval development or function. Could the authors please discuss the egg laying and/or mating effectiveness of the ot1834 allele? Additionally, this could clarify experiments in Figure 2, where the authors appear to be creating new CRISPR alleles for every experiment instead of using traditional mating approaches for a single allele.
5. The authors conclude that deletion of lin-11 eliminates the expression of all "neuron-like" genes. Have the authors tested any non-"neuron-like" gene to ensure the specificity of these effects, as the authors have concluded? If the fate of uv1 cells has changed, how are the authors identifying uv1 cells for analysis in Figure 2A? A panel showing the method for detecting uv1 cells in the wild type and lin-11 mutants would aid in the clarification.

© 2026. Published by The Company of Biologists under the terms of the Creative Commons Attribution License (https://creativecommons.org/licenses/by/4.0/).

6. Comparing mutant alleles to degron-inserted alleles, it's not clear where the differences in the severity of marker expression occurs. Is it due to the loss of uv1 cells or changes in cell placement in genetic mutants, thus impairing the identification of these cells for analyses? Or is it due to genetic mutants eliminating expression of the targeted gene, whereas degron-mediated degradation may not be complete? Have the authors measured the level of degradation of lin-11 or egl-38 they achieve in their system?
7. There is no description of statistical analysis, with the exception of a t-test being performed on data in Figure 3. Statistical analysis was apparently used in Figure 1G and Figure 2B, but no description is provided. Did the authors perform power analysis to determine sample sizes? What p-value was considered the cutoff for statistical significance? Are the number inside the bars on graphs the sample size?

Minor Concerns:
1. The conclusion that lin-11, egl-13, and egl-30 "jointly co-regulate" molecular features of uv1 cells is questionable since mutation or degradation of each gene/protein produces the same phenotype. These findings would suggest necessity of each for target gene expression but not necessarily co-regulation of target genes.
2. The conclusion that lin-11, egl-13, and egl-38 control a "terminal gene battery" is overly simplified based on data showing that lin-11 and egl-13 likely influence fate determination at earlier stages of development.
3. Previously uv1 cells were characterized as neuroendocrine cells. Here they are classified as paraneurons. Could the authors provide a further explanation of the differences in classifications?
4. Using an auxin-inducible degron system, the authors conclude that "LIN-11 is required for the induction/maintenance of the terminal uv1 differentiation program". Have the authors investigated whether the loss of markers in Figure 3A correspond with the appearance of utse markers? Additionally, have the authors used the degron system to probe whether degradation of lin-11, egl-38, or egl-13 at earlier stages of development can reproduce the fate change observed in lin-11 or egl-13 mutants?
5. The orientation and scales of images are not defined. Orientation axes and scale bars would provide a better understanding of the image-based data, especially Figure 1B and C. Additionally, the dotted lines in the fluorescence images are not defined.
6. In Figure S2, what are shown in the right and left panels?
7. The authors conclude that "lin-11, egl-38 and egl-13 therefore define a unique combinatorial signature…" Does this statement pertain to the egg laying apparatus or the entire animal?
8. Figure 1G, Figure 2A, and Figure 4A have graphs with legends to identify "ON" and "OFF". It appears that these designations are identical to labels of "wt" and "lin-11(-)". Could the authors please define the differences and clarify the use of the two labels?
9. The right panel in Figure 1G contains misspellings in the title of the y-axis.
10. In Figure 4A, is the grayscale in the sri-5prom egl-13(-) panel inverted as compared to the other panels?

First revision

<u>Author response to reviewers' comments</u>

Reviewer 1:

SUMMARY OF THE ADVANCE MADE IN THIS PAPER AND ITS POTENTIAL SIGNIFICANCE TO THE FIELD

In this manuscript, the authors document the expression of genes that are expected to be neuronally specific to a set of cells not normally considered neurons in C. elegans. These uv1 cells are part of the egg-laying circuitry and are known to be activated in a mechanosensory fashion. Overall, I find this to be a well written and thoughtful analysis of these cells. I agree with the authors' statement that cells like uv1 and other "paraneurons" do raise intriguing questions about what the definition of a neuron is. In this case, I am curious what the authors think that is. I look at the fact that the cells sense mechanosensory (compression) signals, are secretory, with some

© 2026. Published by The Company of Biologists under the terms of the Creative Commons Attribution License (https://creativecommons.org/licenses/by/4.0/).

(much?) of the exocytotic machinery found in neurons, and these cells facilitate a neuronally-induced behavior (egg-laying). While most neurons in most organisms are largely derived from ectodermal lineages, there are obvious examples of exceptions. In C. elegans there are other neurons derived from the MS lineage, which also generate the uv1 cell types. In addition, the reconstruction of the uv1 cells here demonstrate neuronal-like processes that contain vesicles. Overall, I would encourage the authors to provide a bit more clarity on what separates neurons from paraneurons in the introduction to the paper. Certainly, there are more "secretory" neurons in higher mammals that operate in a fashion similar to uv1, rather than evoked release of chemical neurotransmitters.

I have few concerns about the manuscript. The experiments are well done, appropriate controls are in place, and the quantification, statistical analyses, and presentation are all rigorous. While the overall N number of animals observed in Figure 2 is lower than we might typically see in C. elegans papers, the effects are binary, and thus, highly unlikely to be spurious.

I support this paper for inclusion in Development, specifically I see the advance here as a very thorough genetic dissection of the differentiation of an understudied cell type in C. elegans. The relative question about paraneurons versus neurons should be strengthened to highlight the innovative nature of this, but I believe that can be done with additional information from the authors.

SUGGESTIONS TO AUTHORS

Questions for the authors to address-

1) Can they please be more precise about what features the uv1 cells share with paraneurons that are absent from neurons and/or vice versa? I am not opposed to calling these cells paraneurons, but, it would be helpful to have all of the key facts as we consider this terminology.

Thanks for prompting us to be more precise and explicit about this. All uv1 markers are also expressed in small subset of neurons (further underscoring the case of similarity with neurons). We have clarified this in the text and have also added – as suggested by the reviewer further below – a Table 1 which summarizes the markers, highlighting their neuronal nature. We have also added text in the morphological description of the uv1 neurons that describes why there are not “classic” neurons – with the strongest arguments being the lack of clustered synaptic release sites and their overall unusual morphology.

2) Can they please comment on the perdurance of the egl-3 expression in the HSNs in the CUT triple mutants (this may be relevant to my Q1).

Expression of *egl-3* in HSN appears affected in the CUT triple mutant, but indeed apparently less so in HSN than in uv1. We are not surprised by this because the extent of effects of CUT composite mutants is somewhat different in different cell types within the nervous system and in some cases also requires to removal of all 6 CUT genes. Also, maximal effects on panneuronal genes are usually only achieved upon CUT sextuple removal AND the resident terminal selector(s). We discussed this all in our Leyva-Diaz et al. CUT paper.

3) Can the authors comment on whether any of the auxin depletion experiments resulted in the expression of markers that enabled them to visualize uv1, but led to morphological errors? Specifically, are these terminal selectors responsible for the neuron-like processes that these cells form? It may be possible to see that in an "incomplete" depletion of the protein.

Very good question. We have dealt with this issue a good deal in the context of assessing neuronal functions of terminal selectors. The conundrum in most of these cases – and here with uv1 as well – is that in null mutants the respective cell identity markers are usually off, therefore not allowing a visualization of the respective cell. This is particularly evident here as well. The reviewer is right that one could assess this matter with the temporally controlled depletion approach, and we indeed attempted to assess uv1 morphology using a cytoplasmic marker that is not affected in the depletion experiments. Howver, we observed no obvious defects. Given the clearly hypomorphic effect of the depletion (marker still expressed), this result is difficult to interpret.

Suggestions

I would ask that the authors add the N values for Fig2B and in Figure 3 in the columns as was done for each of the other graphs. I appreciate that the points are added as a scatterplot on top of the bars, but the overlap puts the onus of counting them on the reader.

N values have been added to graphs of what are now Fig.4B and Fig.5

© 2026. Published by The Company of Biologists under the terms of the Creative Commons Attribution License (https://creativecommons.org/licenses/by/4.0/).

Please describe briefly in the text when during development the uv1 cells form, this will be helpful for interpreting the auxin experiments. For example, a graphic timeline of the experiments in Figure 3, that is, when, relative to the formation of the uv1 cells is auxin applied and when are the markers evaluated?
Note, I am not concerned about the "intermediate" effect of depletion, given the genetic loss of function data, but it is worth considering that some earlier events are lin-11 and/or egl-38 dependent and that those events can compensate for later depletion of those proteins.
Yes, these are good points for which we don't have a great answer. In terms of vulval morphogenesis - combined with marker gene expression - the uv1 probably differentiate in mid- to late L4. As we state in the text, this is the time when we treat animals with auxin. One has to consider though that there's inadvertently a delay introduced by the time it takes to absorb enough auxin, then transcription factor degradation to a level below what's necessary and, lastly - and this is probably the most important delay - the time it takes for already produced mRNA and protein product of transcription factor targets to disappear. Of course, we could have simply added auxin earlier, but because of the known effects of these TFs in other cells that produce the EGF signal for uv1 differentiation, we did not want to risk the chance of merely re-demonstrating early induction functions. We therefore left some intentional ambiguity in the text, stating that our experiments show a role in "initiation/maintenance" of terminal differentiation.

I would encourage the authors to present a table of the markers used and the terminal selectors being manipulated (genetically and by auxin). It would be helpful to note the category of proteins that are present.
Thanks again for prompting this, new Table 1 added.

Reviewer 2:

SUMMARY OF THE ADVANCE MADE IN THIS PAPER AND ITS POTENTIAL SIGNIFICANCE TO THE FIELD

The study provides evidence for a set of transcription factors to be involved in cell fate specification of uv1 cells in C. elegans using a battery of cell fate markers. The identified transcription factors are also involved in the specification of neuronal cell fate providing support for the hypothesis that paraneurons and neurons may be sharing evolutionary origins. Therefore, the advance to the field is the description of uv1 cells as paraneurons in the nematode C. elegans and the shared mechanisms of cell fate specification. Overall, this is an interesting concept and contributes to our understanding of the specification of paraneurons in general and specifically in C. elegans. Current shortcomings include the fact that the new findings have not been analyzed or discussed in the context of what is known about cell fate specification of paraneurons more generally.

SUGGESTIONS TO AUTHORS

In this paper Ji and colleagues describe the morphology of the uv1 cells in C elegans, which the authors describe as paraneurons, a term coined in the 70ies. These cells share sets of genes and neuroendocrine functions, but lack the classic morphology of neurons. The authors present an argument that the uv1 cells should be considered paraneurons because they are (1) derived from epithelial cells, (2) sense pressure in the uterus and (3) secrete neuropeptides. The authors then go on to show expression of several cell fate markers (common in neurons), and describe how these cell fate markers are under the control of a set of transcription factors previously known to specify neuronal cell fate. Overall, the study is solid and potentially interesting in that it sheds light on the specification of paraneurons. The manuscript is well written and, strengths include the sophisticated reporters although these reporters do not lead to much different findings than the previously reported reporters. The study also inducible loss of function alleles. These allele show convincingly (what previously loss of function alleles could not) that the tested transcription factor genes are required for the specification of uv1 cells and for maintenance of cell fate. As is, the manuscript reads a bit wormy and no attempt is made to place the findings into a larger context of what is known about the specification of paraneurons more generally. For example, basic HLH are one group of transcriptional regulators that appears to specify paraneurons across phyla [PMID: 41253114], yet no attempt is made to either test or discuss them in the context of the current

© 2026. Published by The Company of Biologists under the terms of the Creative Commons Attribution License (https://creativecommons.org/licenses/by/4.0/).

findings. Placing the current findings into a larger context could significantly increase the appeal to a broader readership.

Points to be addressed:
1. Do mutations in bHLH genes such as lin-32/atoh or others show phenotypes in uv1, either in terms of cell fate or morphology?
This is a good question, given the prominence of bHLH genes in paraneuron-induction in vertebrates. We spent some substantial efforts in addressing this issue. We have reporter gene fusions for all bHLHs. Of all known proneural bHLH genes, we only observed expression in uv1 for *hlh-3*. We found that *hlh-3* null mutants do not affect uv1. However, in parallel, based on ongoing work in the lab – together with some published work from Vincent Bertrand – we know that several bHLH work redundantly. And, somewhat annoyingly, are often expressed at levels below detection (i.e. we have observed proneural defects in neurons that do not show obvious reporter expression). With these two issues in mind, we ignored expression patterns and analyzed three additional proneural bHLHs – lin-32, cnd-1 and ngn-1. Neither single null mutant affected uv1 generation. We then built a quadruple mutant – and, again, no uv1 differentiation defects. However, these quadruple mutants displayed partially penetrant embryonic lethality and the absence of defects in viable escapers it not really interpretable. We also note that another prominent proneural bHLH, hlh-14, could not be examined due to completely penetrant embryonic lethality. We would have loved to use the “Daughterless-trick”, meaning eliminating all bHLH function by removal of the common Daughterless/hlh-2 subunit of bHLHs, but not only are these animals embryonic lethal, but we also know that hlh-2 has important functions right upstream of uv1 generation in vulval patterning. We now mention these bHLH results in the Discussion section.

2. The authors describe a number of similarities between the uv1 paraneurons and neurons in terms of gene expression programs. Where are the differences between paraneurons and neurons in terms of their specification?
This is a really interesting point, thank you for raising this. We know that uv1 requires an inductive EGF signal from neighboring vulF – this is the likely trigger for inducing the proper expression of the uv1 terminal selectors egl-38, lin-11, egl-13 (a point that we now raise in the revised Discussion). EGF is known for some other inductive events in postembryonic development in C. elegans (e.g. it distinguishes the fate of the P11 and P12 neuroblasts), but quite remarkably people have not looked yet how broadly a neurogenic function EGF signaling may have in *C. elegans* neurons. In the heydays of studying EGFR signaling, there were simply not the same set of tools available to probe the effects of EGFR on neuronal identity specification. With them now being available (e.g. in the form of the NeuroPAL ID tool), this is surely fruitful ground for future study. We have added a paragraph in the Discussion to discuss the matter.

3. The authors show that reporters for lin-11, egl-38 and egl-13 are expressed uv1 (as was shown before), leading them to state that "lin-11, egl-38 and egl-13 [] define a unique combinatorial signature of transcription factor[s]". It is not clear why this is a unique signature. Because this combination has never been seen in any other cell?
Yes, correct. Transient co-expression during patterning cannot be excluded but when it comes to marking the terminally differentiated state of a cell, they only overlap in uv1. We know clarify this in the text.

4. At least some of the transcription factor mutants tested show expression of cell fate markers in putative uv1 cells, raising the possibility that in at least a subset of animals uv1 morphology could be investigated using the reporters (such as sri-5 or ida-1).
In the transcription factor genetic mutants tested all uv1 markers analyzed are 100% affected (including sri-5 and ida-1) precluding analysis of uv1 cell morphology.

5. The authors state that CEH-44 and CEH-48 are expressed in uv1. Are the reporters used in Fig.1E transcriptional reporters or protein fusions? Depending on the answer to this question (the strains are not described or referenced), the authors should refer to them as markers for expression of these genes.
These are published, endogenous, CRISPR-engineered GFP tags, now clarified in legend.

6. What is the evidence that the circled cells in Fig.1D-F are uv1 cells? Is position sufficiently unambiguous?

© 2026. Published by The Company of Biologists under the terms of the Creative Commons Attribution License (https://creativecommons.org/licenses/by/4.0/).

In most cases, uv1cells were identified by co-localization with a tdc-1 red marker or an ida-1 red marker in the background, not originally shown in Fig., but now some example shown in a new Figure 3. We have now clarified this in the Figure Legend. We eventually become experienced enough in scoring these cells by position that we did not do it for every single example.

7. p4: should read "First, we assessed expression of the panneuronal marker egl-3…"
Fixed.

8. There are numerous mistakes/omissions in the References
a. Collins et al. 2016 seems duplicated
b. Eroglu et al is incomplete
c. Hills-Muckey et al. is incomplete
d. Sepers et al. is incomplete
e. Young et al. is incomplete
Thanks, will take note of this, it's unfortunately the default output of Zotero when citations have no page number (which many don't have these days). I'll hope that the typesetter can fix this.

Reviewer 3:

SUMMARY OF THE ADVANCE MADE IN THIS PAPER AND ITS POTENTIAL SIGNIFICANCE TO THE FIELD
The manuscript from Ji and colleagues investigated how epithelial cells called uv1 cells develop neuronal features. These uv1 cells were described as neuroendocrine cells in previous studies but are considered paraneuronal cells in this manuscript due to their expression of neuronal genes involved with vesicular release and neuromodulatory functions. Understanding how non-neuronal precursors become bona fide neurons may provide insights into important cellular events that gave rise to neurons during evolution. Studies like these could also shed light onto how the fates of neurons and closely related cells are distinguished in multicellular organisms. To identify how candidate transcription factors influence the expression of neuronal genes in uv1 cells, the authors use fluorescent reporters in animals harboring mutations in three transcription factor genes. Based on these genetic experiments, the authors conclude that lin-11, egl-13, and egl-38 act in a combinatorial manner to coordinate the terminal differentiation of uv1 epithelial cells into paraneuronal cells that express genes associated with neuronal function. Despite excitement for this avenue of research, there are concerns regarding experimental analysis and conclusions drawn from the data provided in this manuscript. The authors identify neuronal genes regulated by these three transcription factors; however, the study provides no mechanistic insight into how they do so. The methods for measuring gene expression lack complete description of the analysis, including statistical analysis and how the authors reliably identified uv1 cells during the studies. The authors focus their conclusions on terminal differentiation. Yet, the importance placed on these events is overstated and over-simplified based on data collected from mutant animals, which display cell fate changes prior to terminal differentiation steps.

Before responding to individually articulated points below, we would like to take the opportunity here to respond to a key criticism of the reviewer – do we overstate or oversimplify our data interpretation. We think that a mere semantic point lies at the core of this criticism. The reviewer introduces here a distinction between "cell fate" and "terminal differentiation". We believe that in this particular case here, the distinction is purely semantic. We now well appreciate from a great number of examples in the literature, both outside worms, but also within worms, that the failure to execute a terminal differentiation program is often accompanied by the execution of an alternative differentiation program. Mechanistically, we also understand in many cases how this works: A terminal selector transcription factor not only activates terminal gene ("effector") batteries but also represses the expression of terminal selectors that drive alternative differentiation programs. Hence, we see no over-simplification in saying that a protein like lin-11 drives a terminal differentiation program, while also being able to repress an alternative differentiation program. The only issue here is whether this is to be called "fate". While we find it not very productive to engage in such a semantic discussion in the manuscript, we do acknowledge that we could have been clearer in describing our phenotypes and their interpretation and we have now added a paragraph in the Discussion to summarize the "fate/identity" phenotype. We thank the reviewer for prompting us to explain our point better.

© 2026. Published by The Company of Biologists under the terms of the Creative Commons Attribution License (https://creativecommons.org/licenses/by/4.0/).

It's also possible that we misunderstand the reviewer's criticism and perhaps the reviewer refers to the earlier function of egl-38 in vulF, which produces the EGF signal required for uv1 induction. It is this early function that we sought to circumvent with our AID experiments. That one and the same factor can have different roles in different cells is of course well appreciated. We also clarify this point now in the Discussion.

SUGGESTIONS TO AUTHORS

Major Concerns:
1. The authors conclude that neuronal genes are expressed in uv1 cells based on fluorescence-based reporters. The data show expression of nuclear localized reporters for genes of interest in a variable number of cells with variable placements within the animals. As presented, it's not clear how the authors identified the uv1 cells in these experiments. The authors show expression patterns of uv1 markers in Figure 1C; however, based on the strain list, these markers were not used to identify uv1 cells for the analyses in this study. How did the authors accurately identify uv1 cells in various mutant and degron expressing backgrounds?
We appreciate the opportunity to clarify this matter: Several images shown in the paper seem to suggest a variability of marker gene expression in cells other than uv1, with variable position. This is simply a reflection of usually showing limited image slices, hence oftentimes other expressing cells are out of focus and therefore not visible in the shown image. This gives a false impression of variability. The scoring of expressed/not expressed was always made with full image stacks. This 3d assessment, in combination with a knowledge of rough uv1 positions, gives us the opportunity to score loss of uv1 expression, as expression in stereotyped other sets of cells (e.g. HSN) is usually maintained. This being said, for the initial description of the wild-type patterns of expression of marker genes, we usually had an ida-1::mCherry marker in the background to confirm uv1 expression. In the degron experiments, the overall patterns of expression remained very similar, with a dimming of expression in a position where the uv1 cells are normally present, giving us again confidence about the correct cell ID.
We now clarified this matter in added paragraphs in the Methods and in several of the figure legends.

2. The authors suggest non-stereotypical vesicles are present in uv1 cells. Based on the EM images, it is difficult to identify the vesicles even with the arrowheads marking two vesicles per cell. More information about the presence of vesicles would help support this conclusion. Do these vesicles appear in multiple sections? In other words, could these images represent cross sections of a larger structure that looks like a vesicle? Do vesicle markers localize to uv1 cells in these same regions? What is the frequency with which vesicles are observed in these cells?
We see vesicles in various places in 27/90 sections we examined, but, no, they are not indications of a larger structure. We clarified this in the legend.

3. The analysis by fluorescence microscopy is incompletely described. What stage(s) of animals were imaged? What are the details of the objective (magnification, immersion, numerical aperture, etc.)?
We have included information about animal stages imaged in the Figure Legends. We have also given more details about objective used for imaging in Materials and Methods section.

4. The authors created a novel null allele of lin-11, since the loss of function effects of previous alleles were deemed questionable. Since previously used loss of function alleles have shown varying effects on the phenotypes, a true null might be expected to present severe defects in vulval development or function. Could the authors please discuss the egg laying and/or mating effectiveness of the ot1834 allele? Additionally, this could clarify experiments in Figure 2, where the authors appear to be creating new CRISPR alleles for every experiment instead of using traditional mating approaches for a single allele.
Yes, all the deletion alleles of *lin-11* that we generated (clean locus deletions) show the exact same pleiotropies of the reference allele, n389: Animals show a 100% penetrant Vul phenotype (hence can't be mated into) and males are exceptionally poor maters (due to male mating defects that we recently described in more detail) – hence, we generated the *lin-11* mutant alleles *de novo* in various reporter backgrounds.

© 2026. Published by The Company of Biologists under the terms of the Creative Commons Attribution License (https://creativecommons.org/licenses/by/4.0/).

5. The authors conclude that deletion of lin-11 eliminates the expression of all "neuron-like" genes. Have the authors tested any non-"neuron-like" gene to ensure the specificity of these effects, as the authors have concluded? If the fate of uv1 cells has changed, how are the authors identifying uv1 cells for analysis in Figure 2A? A panel showing the method for detecting uv1 cells in the wild type and lin-11 mutants would aid in the clarification.
These are again all good points. First of all, we found no markers that are selectively enough expressed in uv1 cells to identify them as such but are not neuronal markers. Hence, in those cases where the uv1 signal is lost, we can only infer such loss from not seeing signal in the region in which uv1 is normally located. Based on the points discussed in #1 above, this is easier than it seems: In 3d image stacks, expression in other cells provides clear enough landmarks to be confident about loss of expression in uv1.

6. Comparing mutant alleles to degron-inserted alleles, it's not clear where the differences in the severity of marker expression occurs. Is it due to the loss of uv1 cells or changes in cell placement in genetic mutants, thus impairing the identification of these cells for analyses? Or is it due to genetic mutants eliminating expression of the targeted gene, whereas degron-mediated degradation may not be complete? Have the authors measured the level of degradation of lin-11 or egl-38 they achieve in their system?
Fluorescently tagged LIN-11 and EGL-38 protein is not visible after auxin treatment indicating a strong depletion. However, we know from past studies of removal of AID-tagged transcription factors in neurons that even if the signal is absent, there's virtually never a recapitulation of the null phenotype, apparently due to residual protein expression below detection level (examples: ceh-34::AID in pharyngeal neurons; unc-86::AID in various sensory neurons; unc-3::AID in motor neurons). Such hypomorphic effects are the most likely explanation, in our opinion, for the partial penetrance and expressivity of the mutant phenotypes.

7. There is no description of statistical analysis, with the exception of a t-test being performed on data in Figure 3. Statistical analysis was apparently used in Figure 1G and Figure 2B, but no description is provided. Did the authors perform power analysis to determine sample sizes? What p-value was considered the cutoff for statistical significance? Are the number inside the bars on graphs the sample size?
We have now included statistical analysis for all data shown in manuscript. Statistical tests used in each figure and P values are listed in figure legends. We have also included a "Statistical analysis" paragraph in the Materials and Methods section.
We did not perform power analysis to determine sample sizes, but we run pilot experiments with at least 5 animals which in many cases already gave statistical significance. $P < 0.05$ was considered to be statistically significant. Yes, number inside bars on graphs are the sample size.

Minor Concerns:
1. The conclusion that lin-11, egl-13, and egl-30 "jointly co-regulate" molecular features of uv1 cells is questionable since mutation or degradation of each gene/protein produces the same phenotype. These findings would suggest necessity of each for target gene expression but not necessarily co-regulation of target genes.
With "joint co-regulation" we mean to say that several transcription factors work together on a target gene to control its expression and that removal of either leads to loss of regulation of this target. In several past precedents, the mechanistic basis for such behavior is either cooperative DNA binding of the transcription factor combination to target DNA, or cooperative recruitment of transcriptional cofactors.

2. The conclusion that lin-11, egl-13, and egl-38 control a "terminal gene battery" is overly simplified based on data showing that lin-11 and egl-13 likely influence fate determination at earlier stages of development.
Please note our more extensive response to this point earlier on.
Let me re-iterate: The reviewer is either referring to what we think is – in this particular case – a semantic distinction between "fate" and "terminal differentiation. Or the reviewer refers with "fate" to the earlier roles that egl-38 may play in vulF specification. In the latter case, yes, if we had only looked at the null mutant phenotypes, then, yes, the effect could be explained by fate induction from vulF at earlier stages of development. But since we find that removal of at least LIN-11 and EGL-38 AFTER the initial vulF-mediated fate determination step also affects uv1 (AID experiments), this suggests a role in terminal differentiation, rather than just early fate

© 2026. Published by The Company of Biologists under the terms of the Creative Commons Attribution License (https://creativecommons.org/licenses/by/4.0/).

determination. Early and late functions of transcription factors, even in the same lineage, are broadly appreciated (e.g. unc-86) and one does not exclude the other.

3. Previously uv1 cells were characterized as neuroendocrine cells. Here they are classified as paraneurons. Could the authors provide a further explanation of the differences in classifications?
This is not a contradiction. Many neuroendocrine cells are paraneurons, but what classifies uv1 specifically as paraneurons is that they also have an interoceptive function. We have discussed this extensively in a recent piece in Curr Biol to which we refer to here. We now clarify this in the introduction.

4. Using an auxin-inducible degron system, the authors conclude that "LIN-11 is required for the induction/maintenance of the terminal uv1 differentiation program". Have the authors investigated whether the loss of markers in Figure 3A correspond with the appearance of utse markers? Additionally, have the authors used the degron system to probe whether degradation of lin-11, egl-38, or egl-13 at earlier stages of development can reproduce the fate change observed in lin-11 or egl-13 mutants?
We did not score uv1 markers and utse markers in the same animals, but generally, yes, we see a gain of utse markers and a loss of uv1 markers in lin-11 animals.
Yes, we have removed lin-11 earlier and this clearly does NOT fully recapitulate the 100% penetrant Vul phenotype observed in lin-11 null mutants. We have consistently seen that the removal of postmitotically acting fate-defining transcription factors, results in a hypomorphic effect – as we do see here in the uv1 cells, where the L4-onward removal does not lead to as strong a phenotype as the null phenotype.

5. The orientation and scales of images are not defined. Orientation axes and scale bars would provide a better understanding of the image-based data, especially Figure 1B and C. Additionally, the dotted lines in the fluorescence images are not defined.
Apologies for the oversight. We have added orientation axes to Fig.1 and have clarified image orientation in Figure Legends of all other figures. Scale bars have been added to all images. Dotted lines have been defined in Figure Legends.

6. In Figure S2, what are shown in the right and left panels?
We have added labels to all panels (now main Figure 3)

7. The authors conclude that "lin-11, egl-38 and egl-13 therefore define a unique combinatorial signature…" Does this statement pertain to the egg laying apparatus or the entire animal?
Entire animal, at least in terminally differentiating/differentiated cells. In those, only uv1 shows an overlap of expression of these 3 transcription factors. Now clarified in text.

8. Figure 1G, Figure 2A, and Figure 4A have graphs with legends to identify "ON" and "OFF". It appears that these designations are identical to labels of "wt" and "lin-11(-)". Could the authors please define the differences and clarify the use of the two labels?
Labels "wt" and "lin-11(-)" indicate whether the picture or graph column shows a wild type or lin-11 mutant animal. The labels "ON" and "OFF" indicate whether the specific reporter being analyzed is expressed (ON) or not expressed (OFF) in wild type or mutant animals. Now clarified in legend.

9. The right panel in Figure 1G contains misspellings in the title of the y-axis.
We have corrected typo on the y-axis.

10. In Figure 4A, is the grayscale in the sri-5prom egl-13(-) panel inverted as compared to the other panels?
No, grayscale in sri-5Prom egl-13(-) image is the same as other images (black signal on a light background).

Second decision letter

© 2026. Published by The Company of Biologists under the terms of the Creative Commons Attribution License (https://creativecommons.org/licenses/by/4.0/).

MS ID#: dev.205749R1

MS TITLE: Regulatory architecture controlling terminal differentiation of an interoceptive paraneuron in C. elegans

AUTHORS: Hongzhu Ji; Berta Vidal; Elisabeth Conklin; Tergel Enkhtuvshin; Nathan E. Schroeder; Oliver Hobert

Dear Dr Hobert,

I am happy to tell you that your manuscript has been accepted for publication in Development, pending our standard publication integrity checks.

Reviewer 1

SUMMARY OF THE ADVANCE MADE IN THIS PAPER AND ITS POTENTIAL SIGNIFICANCE TO THE FIELD

The paper describes an attempt to understand the contributions of different transcription factors to the uv1 cells in C. elegans. These are described in the literature as neuroendocrine cells, but with new EM analysis and neuronal marker analysis, these authors advocate for the term "paraneuron." As in the first version, the advancement here is the rigorous assessment of the consequence of different transcription factors to the differentiation of these cells. They use a combination of existing genetic alleles, generated new genetic alleles, and used conditional depletion. The study relies on multiple distinct markers for the cell type and for neurons in general. The authors raise the question, are paraneurons potentially evolutionary precursors to neurons? To more adequately answer that question we need to know if paraneurons exhibit the same kinds of disruptions to specification as neurons. This article suggests that they do in fact act similarly to neurons in how discrete features are specified by a combination of panneuronal TFs and terminal selectors. I think this will help the field think about how neurons may have adapted from secretory cells that did not form classic synapses into our more modern definition.

SUGGESTIONS TO AUTHORS

Reviewer 2

SUMMARY OF THE ADVANCE MADE IN THIS PAPER AND ITS POTENTIAL SIGNIFICANCE TO THE FIELD

as before

SUGGESTIONS TO AUTHORS

The authors have addressed by previous comments/concerns satisfactorily. I have found a couple of typos that the authors may want to correct:
Abstract, 3rd line; "neuron-like features"
Intro, 4th last line: "analysis not only provide" -> should be "analyses not only provide" OR "analysis not only provides"

© 2026. Published by The Company of Biologists under the terms of the Creative Commons Attribution License (https://creativecommons.org/licenses/by/4.0/).
